# Validation of a fibrinogen γ’ enzyme-linked immunosorbent assay using a new monoclonal antibody: effects of bariatric surgery

**DOI:** 10.1016/j.rpth.2024.102319

**Published:** 2024-01-17

**Authors:** Nadja Bødker Pedersen, Else-Marie Bladbjerg, Claus Bogh Juhl, Anette Larsen, Anna-Marie Bloch Münster, Moniek P.M. de Maat, Yaseelan Palarasah

**Affiliations:** 1 Unit for Thrombosis Research, Department of Clinical Biochemistry, University Hospital of Southern Denmark, Esbjerg, Denmark; 2 Department of Regional Health Research, University of Southern Denmark, Denmark; 3 Department of Medicine, Section of Endocrinology, University Hospital of Southern Denmark, Esbjerg, Denmark; 4 Steno Diabetes Center Odense, Odense, Denmark; 5 Department of Hematology, Erasmus MC, University Medical Center Rotterdam, Rotterdam, The Netherlands; 6 Department of Cancer and Inflammation Research, University of Southern Denmark, Odense, Denmark

**Keywords:** antibodies, monoclonal, bariatric surgery, enzyme-linked immunosorbent assay, fibrinogen, obesity

## Abstract

**Background:**

Fibrinogen γ’ is a naturally occurring 20-amino-acid splice variant of the fibrinogen γ chain. Animal studies link variations in fibrinogen to obesity, but it is unknown how fibrinogen γ’ is associated with obesity in humans.

**Objectives:**

To develop and validate an enzyme-linked immunosorbent assay (ELISA) for fibrinogen γ’ quantification in human plasma and analyze fibrinogen γ’ before and after bariatric surgery.

**Methods:**

We generated C-terminal fibrinogen γ’ specific mouse monoclonal antibodies and developed a γ’ ELISA. Validation included measures of accuracy, sensitivity, and precision. Fibrinogen γ’ and total fibrinogen were measured in 60 individuals before and 6 months after bariatric surgery and in 19 normal-weight controls and 120 blood donors.

**Results:**

Highly specific fibrinogen γ’ monoclonal antibodies were produced and successfully used in the ELISA. Recovery was 88%, and limits of detection and quantification were 0.003 mg/mL and 0.014 mg/mL, respectively. Coefficients of variation were 3% for repeatability and 7% for within-laboratory variation. The fibrinogen γ’ reference interval was 0.25 to 0.80 mg/mL. Fibrinogen γ’ concentrations were reduced after bariatric surgery and were higher in individuals with obesity than in those with normal weight. The fibrinogen γ’/total fibrinogen ratio was unchanged after surgery but was higher than the ratio in normal-weight individuals.

**Conclusion:**

We developed a precise and sensitive ELISA for fibrinogen γ’. Levels of fibrinogen γ’, but not the fibrinogen γ’/fibrinogen ratio, were reduced 6 months after bariatric surgery. Absolute and relative levels of fibrinogen γ’ were increased in individuals with obesity compared to normal-weight individuals.

## Essentials

• Animal studies link variants of the plasma protein fibrinogen to obesity.
• We developed a precise and sensitive enzyme-linked immunosorbent assay for detection of the variant fibrinogen γ’.
• We used the enzyme-linked immunosorbent assay in 60 patients before and after bariatric surgery and in normal-weight persons.
• Fibrinogen γ’ levels decreased after bariatric surgery and were lower in normal-weight persons.

## 1 Introduction

Fibrinogen is a central protein in the coagulation cascade and has a well-documented association with risk of cardiovascular disease (CVD) [[1], [2], [3]]. In humans, there are large numbers of genetic variations and posttranslational modifications in fibrinogen [4,5]. A common, naturally occurring variant of the fibrinogen γ chain, fibrinogen γ’, was discovered in 1972 [6], and this chain has a higher molecular weight (51.5 kDa) than the common γA chain (49.5 kDa). Fibrinogen γ’ is reported to account for 12% of total fibrinogen in blood [6]. It is generated as a result of alternative splicing and polyadenylation at site 1 of the ninth intron of the *FGG* gene. This leads to the creation of a new C-terminal, where the last 4 residues (AGDV) of the γA chain are replaced by a unique 20-amino-acid sequence (VRPEHPAETEYDSLYPEDDL) [[7], [8], [9]]. The amino acid residues within the fibrinogen γ’ sequence give the extended C-terminal a negative charge, exerting distinct effects on fibrin formation compared with the predominant γA chain.

Biochemical properties of fibrin formed from the heterozygous form, fibrinogen γA/γ’, vary from those of the common isoform γA/γA. It has been shown that γA/γ’ fibrin has a reduced fiber diameter and pore size as well as increased branching [10]. Hence, the fibrin network of clots made of fibrinogen γA/γ’ is finer and more branched than the fibrin network of γA/γA, which might be caused by the slower release of fibrinopeptide B from fibrinogen γA/γ’ [11]. Another difference between the 2 isoforms is the increased resistance to lysis of γA/γ’ fibrin clots, likely explained by increased crosslinking induced by the enhanced interaction of γA/γ’ fibrinogen with factor (F)XIII [[11], [12], [13]]. The negatively charged C-terminal creates high-affinity binding sites for FXIII [13] and thrombin [14], and the high-affinity interaction of fibrinogen γA/γ’ with thrombin leads to a relative protection of thrombin against inhibition by antithrombin [15].

In keeping with these changes in fibrin clot architecture, several studies have shown that increased levels of fibrinogen γ’ are associated with arterial thrombosis. In these studies, it was demonstrated that the levels of fibrinogen γ’ are increased in patients with peripheral arterial disease [16], coronary artery disease [17], myocardial infarction [18], and ischemic stroke [19,20]. This was accompanied by increased [16,17,19], decreased [21], or unchanged [18] ratio of fibrinogen γ’ to total fibrinogen. Further, a significant positive association between fibrinogen γ’ concentrations and cardiovascular risk factors is observed, also when adjusting for the concentration of total fibrinogen [21,22]. In contrast, studies have suggested a negative association between fibrinogen γ’ levels and venous thrombosis [[23], [24], [25]].

A study in mice has linked variations in fibrinogen to obesity [26], but it is unknown how fibrinogen γ’ is associated with obesity in humans. A small study has observed enhanced fibrinogen γ’ concentrations in 15 children with obesity compared with 6 normal-weight children. A minor decrease in fibrinogen γ’ levels was observed after lifestyle-induced weight loss [27], although the ratio of fibrinogen γ’ to total fibrinogen was similar between children with obesity and those with normal weight and was unchanged after weight loss. Larger studies are, however, needed to understand more about the association between obesity and fibrinogen γ’. The aim of the present study was to develop and validate a fibrinogen γ’ enzyme-linked immunosorbent assay **(**ELISA) and apply the assay to clinical samples comparing fibrinogen γ’ in individuals before and 6 months after bariatric surgery.

## 2 Methods

### 2.1 Buffers for ELISA

Buffers for ELISA included a coating buffer (15 mM Na_2_CO_3_, 35 mM NaHCO_3_, pH 9.5), washing buffer (135 mM NaCl, 5 mM Na_2_HPO_4_ [PanReac Applichem], 1.5 mM NaH_2_PO_4_, 2.7 mM KCl, 0.05% Tween 20 [VWR Chemicals], pH 7.4), and blocking/dilution buffer (washing buffer with 0.1% [v/v] bovine serum albumin). Unless otherwise stated, reagents were obtained from Merck.

### 2.2 Buffers for sodium dodecyl sulfate-polyacrylamide gel electrophoresis and western blot

Buffers included running buffer (20X MES SDS Running Buffer [Invitrogen] diluted to 5% in demineralized water), sample buffer (NuPAGE LDS Sample Buffer [4X], Invitrogen), transfer buffer (10 mL of 20X transfer buffer [Invitrogen] diluted to 5% in demineralized water and 20% [v/v] of 98% ethanol, pH 8.4), blocking/washing buffer (135 mM NaCl, 5 mM Na_2_HPO_4_ [PanReac Applichem], 1.5 mM NaH_2_PO_4_, 2.7 mM KCl, 0.05% Tween 20 [VWR Chemicals], pH 7.4), and sodium acetate buffer (70 mM CH_3_COONa [PanReac Applichem], 30 mM CH_3_COOH, pH 5.0).

### 2.3 Generation of anti-fibrinogen γ’ monoclonal antibodies

Monoclonal antibodies (mAbs) specific for the C-terminal fibrinogen γ’ chain were generated by immunizing Naval Medical Research Institute mice with a synthetic peptide corresponding to the fibrinogen γ’ 20 amino acid peptide C-VRPEHPAETEYDSLYPEDDL with additional N-terminal Cys residue used for coupling to diphtheria toxoid [28]. The mice were immunized twice subcutaneously, at an interval of 14 days, with 20 μg of the coupled synthetic peptide mixed with GERBU Adjuvants (GERBU Biotechnik GmbH). Fourteen days after the last injection and 3 days prior to fusion, the mice were immunized intravenously with the coupled peptide, and spleen cells were recovered and fused with myeloma cells, following the principles of Köhler and Milstein [29]. Supernatants from hybridomas were initially screened using Maxisorb ELISA plates (Thermo Fisher Scientific) coated with 0.5 μg/mL purified fibrinogen (Sigma Aldrich), followed by at least 3 rounds of cloning by limited dilution, ensuring clonality of the final hybridoma. Single clones were then grown in culture flasks in RPMI-1640 (Lonza, BioWhittaker) supplemented with sodium pyruvate (Gibco) and gentamicin sulfate (Biowest) containing 10% fetal bovine serum (Biowest).

### 2.4 Sodium dodecyl sulfate-polyacrylamide gel electrophoresis and western blot analysis

The mAb specificity toward fibrinogen γ’ was evaluated and compared with polyclonal rabbit antihuman fibrinogen (Dako) by sodium dodecyl sulfate-polyacrylamide gel electrophoresis (SDS-PAGE). Plasma, fibrinogen γA (P1 Fib, Enzyme Research Laboratories), fibrinogen γ’ (P2 Fib, Enzyme Research Laboratories), and serum (negative control) were used as sources of fibrinogen. Proteins were separated by SDS-PAGE in gels (Invitrogen) and transferred to polyvinylidene fluoride membranes (GE Healthcare Bio-sciences AB) using standard methods. Membranes were blocked with blocking buffer overnight at room temperature (RT) and incubated with 1 μg/mL mouse anti-fibrinogen γ’ mAb or polyclonal rabbit antihuman fibrinogen (Dako) and stirring for 1 hour. The membranes were washed thrice in washing buffer and incubated for 1 hour at RT with a 1:1000 dilution of horseradish peroxidase (HRP)–conjugated polyclonal rabbit antimouse immunoglobulin G antibody (Invitrogen) or HRP-conjugated swine antirabbit immunoglobulin G antibody (Dako). The membranes were developed using 0.4 mg/mL 3-amino-9-ethylcarbazole (Sigma Aldrich) in 50 mM sodium acetate buffer with 0.015% H_2_O_2_ (Sigma Aldrich).

### 2.5 Plasma for ELISA development and validation

Healthy individuals provided blood samples and gave oral and written informed consent. Sampling was conducted according to the Declaration of Helsinki. Platelet-poor plasma was prepared immediately after sampling, and tubes for serum were left at 20 °C for 30 minutes before centrifugation for 20 minutes at 2000 ×*g* (20 °C). Plasma and serum were rapidly frozen and stored at −80 °C until analyzed. All samples were analyzed in duplicate.

#### 2.5.1 Plasma and serum pools

Plasma was collected in trisodium citrate (0.109 M Na_3_-citrate, Becton Dickinson, reference: 363048) and EDTA (Becton Dickinson, reference: 365900) tubes, and serum was collected in clot activator tubes (Becton Dickinson, reference: 369032) from 20 healthy individuals at the Department of Clinical Biochemistry, University Hospital of Southern Denmark, Esbjerg, Denmark. These samples were pooled and used in all experiments during the setup and validation of the fibrinogen γ’ ELISA.

#### 2.5.2 Reference material

Plasma was collected in citrate tubes (Becton Dickinson, reference: 363048) from 120 healthy blood donors (60 women and 60 men; mean age, 30 years; mean body mass index [BMI], 25.6 kg/m^2^; no use of oral contraceptives and medicine). These samples were used to define the reference interval of the ELISA, given as fibrinogen γ’ and as the ratio of fibrinogen γ’ to total fibrinogen.

### 2.6 ELISA for measurement of fibrinogen γ’

The fibrinogen γ’ assay was constructed as a noncompetitive sandwich ELISA using fibrinogen γ’ mAb as capture antibody and biotinylated polyclonal rabbit antihuman fibrinogen as detection antibody.

Maxisorp ELISA plates (Thermo Fisher Scientific) were coated with 2 μg/mL fibrinogen γ’ mAb in coating buffer overnight at 4 °C in a humidity chamber. Plates were washed 4 times in washing buffer and blocked for 20 minutes in blocking buffer. Samples, calibrators, and controls were diluted in dilution buffer and incubated for 1 hour at RT with stirring, followed by washing 4 times in washing buffer. Biotinylated polyclonal rabbit antihuman fibrinogen was added in 1:500 dilution and incubated for 1 hour at RT with stirring. Following washing for 4 times, HRP-conjugated streptavidin (Invitrogen) diluted to 1:3000 was added and incubated with stirring for 30 minutes at RT. Plates were washed 4 times and visualized by incubating with 3,3’,5,5’-tetramethylbenzidine staining substrate (ECO-TEK Kementec) for 15 minutes protected by light. Next, color development was terminated by adding 0.2 M H_2_SO_4_. Finally, the plates were read at 450 nm, with 650 nm as a reference, using a Sunrise absorbance microplate reader (Tecan Group Ltd).

#### 2.6.1 Assay calibration

A 7-point calibration curve of fibrinogen γ’ (P2 Fib) from 16 ng/mL to 1000 ng/mL was constructed to analyze the amount of fibrinogen γ’ in plasma samples. Citrated plasma samples were diluted 1:20,000 (v/v) in dilution buffer and analyzed in the ELISA. Plasma levels of fibrinogen γ’ were calculated using a 4-parameter nonlinear logistic curve fitting (Magellan Data Analysis Software v. pro 7.3 for PC, Tecan Group Ltd).

### 2.7 Assay validation

#### 2.7.1 Parallelism

Parallelism was investigated to demonstrate that the calibrator and the analyte are recognized equally well in the assay [30]. Serial dilutions of the citrated plasma pool and calibrator were investigated for parallelism. Both the calibrator and the citrated plasma pool were diluted in the dilution buffer and analyzed in duplicate.

#### 2.7.2 Spike and recovery

The purpose of a spike and recovery test is to ensure that any differences between sample matrix and calibrator diluent do not affect the response signal [30]. Citrated plasma pool was spiked with 0.225 mg/mL of fibrinogen γ’ (P2 Fib) (*n* = 20) or dilution buffer (*n* = 20) and diluted 1:20,000 in dilution buffer. The levels of fibrinogen γ’ were measured using the fibrinogen γ’ ELISA, and the concentration of fibrinogen γ’ in the nonspiked citrated plasma pool was subtracted to calculate the recovery percentage.

#### 2.7.3 Limit of detection, limit of quantification, and precision estimates

Limit of detection (LOD) and limit of quantitation (LOQ) are parameters used to describe the smallest measurable concentrations of the analytical procedure. LOD is the lowest analyte concentration that can be separated from the background signal of the assay, while LOQ is the lowest analyte concentration that can be quantitated at some defined levels, taking imprecision and accuracy into account [31].

The minimum asymptote of the 4-parameter nonlinear logistic curve fitting represents the minimum value that can be obtained, thus corresponding to the value of a blank sample. Based on the minimum asymptote, LOD was defined as the minimum asymptote + 2 SD, while LOQ was defined as the minimum asymptote + 10 SD [32]. The SD value was calculated from 10 samples of fibrinogen-depleted plasma (Affinity Biologicals) diluted 1:20,000 and analyzed in the fibrinogen γ’ ELISA.

The guidelines from the Clinical and Laboratory Standards Institute document EP05-A2 were used [33] to evaluate the precision of the method. The fibrinogen γ’ concentration was analyzed in citrated plasma at 2 dilutions, 1:10,000 (low level) and 1:35,000 (high level), and in Standard Human Plasma (Siemens Healthcare Diagnostics Products GmbH) diluted 1:20,000 (decision-point level). The procedure was repeated twice a day for 20 days, and the estimate of repeatability SD (S_r_) and the total within-laboratory SD (S_T_) were calculated. The relative SDs were expressed as the coefficients of variation (CV).

#### 2.7.4 Freezing and thawing

Citrated samples that were thawed once (*n* = 10) were compared with citrated samples that were thawed twice (*n* = 10), thrice (*n* = 10), and 4 times (*n* = 10).

#### 2.7.5 Citrate plasma vs EDTA plasma

Citrated pool samples (*n* = 20) were compared with EDTA pool samples (*n* = 20). The concentration of fibrinogen γ’ in the citrated plasma samples was adjusted for the dilution factor of 17%, made up of the citrate solution added to whole blood.

#### 2.7.6 Reference interval

Citrated plasma samples from 120 healthy blood donors (60 men and 60 women) from the Department of Clinical Immunology, University Hospital of Southern Denmark, Esbjerg, Denmark, with a mean (SD) age of 29.8 (10.2) years and a mean (SD) BMI of 25.6 (5.1) kg/m^2^, were analyzed using the fibrinogen γ’ ELISA. Concentrations of total fibrinogen were determined with fibrinogen antibodies (Siemens Healthcare Diagnostics Products GmbH) using the BN II analyzer (Siemens Healthcare Diagnostics Products GmbH). The results describe the reference interval of the fibrinogen γ’ ELISA and were calculated as mean fibrinogen γ’ concentration ± 2 SD and mean fibrinogen γ’/fibrinogen ratio ± 2 SD.

### 2.8 Fibrinogen γ’ before and after bariatric surgery

We applied the fibrinogen γ’ ELISA in a clinical study of individuals with severe obesity before and 6 months after bariatric surgery.

#### 2.8.1 Study design and participants

This research is part of a study investigating the effects of bariatric surgery followed by physical training, and the study design, patient recruitment, and trial registration have been described in details elsewhere [34]. In brief, we included 42 ethnic Danish women and 18 ethnic Danish men with a mean age of 42.3 years and a mean BMI of 43.0 kg/m^2^ who were eligible for Roux-en-Y-gastric bypass (RYGB) according to national guidelines (BMI > 35 kg/m^2^ with obesity-related diseases or BMI > 50 kg/m^2^ with obesity-related social or physical complications). Major medications were antihypertensives (*n* = 27), antidiabetic agents (*n* = 18), and lipid-lowering drugs (*n* = 11) to treat the major comorbidities of hypertension, type II diabetes, and dyslipidemia. Nineteen patients did not receive any medications. Smoking patients and patients receiving anticoagulant medications or hormone replacement therapy/oral contraceptives were excluded. The patients underwent RYGB at the Department of Surgery, University Hospital of Southern Denmark. Blood samples for the present study were obtained before and 6 months after surgery.

Nineteen healthy and normal-weight individuals from the Department of Clinical Biochemistry, University Hospital of Southern Denmark, were matched in terms of age and sex assigned at birth with the 19 medication-free patients used as a control group.

#### 2.8.2 Blood sampling and analysis

Venous blood samples were collected in the morning after an overnight fast and 15 minutes of rest in the supine position. Samples were collected in citrate tubes (Becton Dickinson, reference: 363048), and platelet-poor plasma was prepared by centrifugation at 2000 ×*g* (20 °C) for 20 minutes. Plasma samples were stored at −80 °C until analyzed in 1 series for each patient before and after RYGB. Samples were analyzed for fibrinogen γ’ and fibrinogen.

### 2.9 Statistical analysis

GraphPad Prism version 9.3.1 (GraphPad Software) was used for all statistical analyses. Concentrations of fibrinogen γ’ in citrated and EDTA plasma were compared with the Mann–Whitney U-test, and levels in citrated plasma thawed 1, 2, 3, and 4 times were compared with the Friedman test. To investigate parallelism, an analysis of covariance was used to test equality of regression lines.

Concentrations of fibrinogen and fibrinogen γ’ in the clinical and reference samples were normally distributed. In the reference samples, women and men, as well as age groups (<40 years vs >40 years), were compared with an unpaired *t*-test. Results before and after RYGB were compared with a paired *t*-test for all patients (*n* = 60) and medication-free patients (*n* = 19). Medication-free patients before surgery and normal-weight individuals (*n* = 19) were also compared using a paired *t*-test, as each person was matched in terms of age and sex. Correlations were determined using the Pearson correlation coefficient (*r*).

A *P* value of <.05 was considered significant. The results are presented as median and IQR, mean and SD, or mean and 95% CIs.

## 3 Results

### 3.1 Specificity of antihuman fibrinogen γ’ monoclonal antibodies 19-5-1

In total, 6 mAbs producing hybridoma cells were obtained, and based on the initial screening in relation to reactivity and background, clone 19-5-1 was selected for assay development (results not shown).

**Figure 1:**
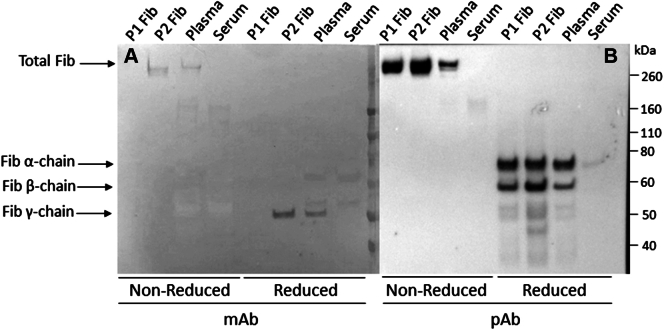
Western blot of mouse anti-fibrinogen γ’ monoclonal antibody (mAb) 19-5-1 in nonreduced and reduced form (A) and rabbit anti-fibrinogen polyclonal antibody (pAb) in nonreduced and reduced form (B). Fib, fibrinogen; P1 Fib, fibrinogen γA; P2 Fib, fibrinogen γ’.

Western blot analysis using mAb 19-5-1 (Figure 1A) demonstrated visible bands for plasma and fibrinogen γ’ (P2 Fib) with apparent molecular weight of 340 kDa, corresponding to the size of the intact fibrinogen molecule in the lanes of nonreduced samples. In the lanes of the reduced samples, only clear and distinct bands at 50 kDa were detected, corresponding to the molecular weight of fibrinogen γ’. For fibrinogen γA (P1 Fib), no bands were detectable when blotting with the mAb 19-5-1. Blotting analysis using polyclonal rabbit anti-fibrinogen antibody (Figure 1B) resulted in bands at 340 kDa in the nonreduced samples of fibrinogen γA (P1 Fib), fibrinogen γ’ (P2 Fib), and plasma, whereas bands at 68 kDa, 55 kDa, and 49 kDa were seen in the reduced samples corresponding to the α, β, and γ chain of fibrinogen, respectively. No bands were detected using serum as a negative control. These results show that the developed mouse anti-fibrinogen γ’ mAb 19-5-1 only reacts with specificity toward the γ’ chain.

**Figure 2:**
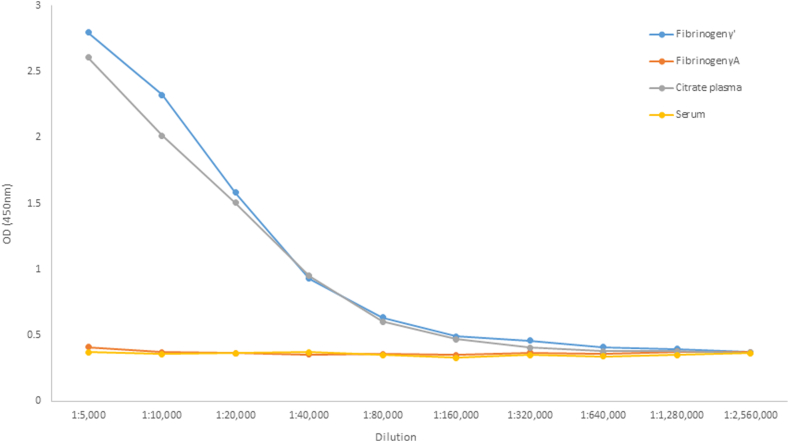
Serial dilutions of fibrinogen γ’ (P2 Fib), fibrinogen γA (P1 Fib), citrated plasma pool, and serum pool in dilution buffer using the fibrinogen γ’ ELISA. OD, optical density.

Based on these results, specificity of mAb 19-5-1 was further analyzed in the ELISA. Using mAb 19-5-1 as capture antibody, no signal for fibrinogen γA (P1 Fib) was obtained. Fibrinogen γ’ (P2 Fib) and citrated plasma pool resulted in dose-dependent signals (Figure 2).

### 3.2 Assay validation

#### 3.2.1 Parallelism

Parallelism was observed between serial dilutions of fibrinogen γ’ (P2 Fib) and citrated plasma pool (Figure 2). Analysis of covariance showed no significant differences (*P* = .99) between slopes of the regression lines.

#### 3.2.2 Spike and recovery

Citrated plasma (*n* = 20) was spiked with 0.225 mg/mL of fibrinogen γ’ (P2 Fib) or dilution buffer (*n* = 20). The mean recovery percentage of fibrinogen γ’ was 88% (95% CI, 75%-102%).

#### 3.2.3 LOD, LOQ, and precision estimates

LOD and LOQ for the fibrinogen γ’ ELISA were 0.003 mg/mL and 0.014 mg/mL, respectively. The precision estimate for the level of decision-point was found to be 0.015 mg/mL, expressed as a CV of 3% for the repeatability SD estimate S_r_, whereas the within-laboratory SD estimate S_T_ was 0.038 mg/mL, expressed as a CV of 7%. For the low level, S_r_ was 0.022 mg/mL, expressed as a CV of 4%, while S_T_ was 0.076 mg/mL, expressed as a CV of 13%. For the high level, S_r_ was 0.019 mg/mL, expressed as a CV of 3%, whereas S_T_ was 0.070 mg/mL, expressed as a CV of 10%.

#### 3.2.4 Freezing and thawing

We observed no difference (*P* = .90) in median fibrinogen γ’ concentrations between citrated plasma samples (*n* = 10) that were thawed 1 (0.63 [IQR, 0.60-0.66] mg/mL), 2 (0.63 [IQR, 0.60-0.68] mg/mL), 3 (0.66 [IQR, 0.61-0.71] mg/mL), and 4 times (0.65 [IQR, 0.55-0.71] mg/mL).

#### 3.2.5 Citrate plasma vs EDTA plasma

We observed no difference (*P* = .09) in median fibrinogen γ’ concentrations in 20 citrated plasma pool samples (0.69 [IQR, 0.65-0.72] mg/mL) and 20 EDTA plasma pool samples (0.72 [IQR, 0.65-0.77] mg/mL).

#### 3.2.6 Reference interval

The mean plasma concentration of fibrinogen γ’ was 0.52 (SD 0.14) mg/mL, yielding a reference interval of 0.25 to 0.80 mg/mL (*n* = 120). For females (*n* = 60) and males (*n* = 60), the level of fibrinogen γ’ was 0.53 (SD 0.13) and 0.51 (SD 0.15) mg/mL, respectively, with no difference between the 2 sexes (*P* = .51). Also, no differences were seen between individuals aged >40 years (0.49 [SD 0.14] mg/mL, *n* = 19) and <40 years (0.53 [SD 0.14] mg/mL, *n* = 101) (*P* = .27).

The mean plasma concentration of total fibrinogen was 2.81 (SD 0.56) mg/mL, resulting in a reference interval of 1.68 to 3.94 mg/mL. This led to a mean fibrinogen γ’/fibrinogen ratio of 0.19 (SD 0.05) and a reference interval of 0.09 to 0.29. A positive correlation was observed between fibrinogen and fibrinogen γ’ (*r* = .41; *P* < .001). As for total fibrinogen γ’ levels, no difference was observed between sex or age groups (<40 years vs >40 years) for fibrinogen γ’/fibrinogen ratio (results not shown).

### 3.3 Fibrinogen γ’ before and after bariatric surgery

Measures of body weight and fibrinogen before and 6 months after bariatric surgery are presented in Table 1. The results show a significant reduction in BMI, fibrinogen γ’, and fibrinogen, whereas the ratio of fibrinogen γ’ to fibrinogen did not change significantly after the surgery. Correlations were observed between fibrinogen γ’ and fibrinogen before (*r* = .48; *P* < .001) and after (*r* = .54; *P* < .001) surgery and between surgery-induced changes (*r* = .55; *P* < .001). There were no correlations between BMI and fibrinogen γ’ before (*r* = .19; *P* = .14) and after (*r* = .17; *P* = .19) surgery and between surgery-induced changes (*r* = −.14; *P* = .29) and between BMI and the fibrinogen γ’/fibrinogen ratio before (*r* = −.01; *P* = .93) and after (*r* = .01; *P* = .93) surgery and between surgery-induced changes (*r* = −.16; *P* = .23).

**Table 1:** Body mass index and fibrinogen measures before and 6 months after bariatric surgery.

Variable	Before surgery (*n* = 60)	After surgery (*n* = 60)	*P* values
BMI (kg/m^2^)	43.0 (41.4-44.5)	33.7 (32.2-35.2)	<.001
Fibrinogen γ’ (mg/mL)	1.32 (1.21-1.42)	1.22 (1.13-1.30)	.03
Fibrinogen (mg/mL)	4.16 (3.95-4.37)	3.69 (3.49-3.89)	<.0001
Fibrinogen γ’/fibrinogen	0.32 (0.30-0.34)	0.33 (0.31-0.35)	.08

Mean values (95% CIs) before and 6 months after surgery were compared with a paired *t*-test.

BMI, body mass index.

The reductions in BMI and fibrinogen after surgery as well as the unchanged ratio between fibrinogen γ’ and fibrinogen were confirmed in 19 medication-free individuals. However, the reduction in fibrinogen γ’ was not significant (*P* = .06) (Table 2).

**Table 2:** Body mass index and fibrinogen measures before and 6 months after bariatric surgery in medication-free individuals and matched normal-weight controls.

Variable	Before surgery (*n* = 19)	After surgery (*n* = 19)^a^	*P* values^a^	Controls (*n* = 19)^b^	*P* values^b^
BMI (kg/m^2^)	44.8 (41.6-47.9)	35.0 (32.0-38.0)	<.0005	22.5 (21.7-23.3)	<.0005
Fibrinogen γ’ (mg/mL)	1.33 (1.13-1.52)	1.22 (1.04-1.39)	.06	0.76 (0.67-0.84)	<.0001
Fibrinogen (mg/mL)	4.31 (3.89-4.73)	3.85 (3.51-4.19)	<.0001	3.13 (2.87-3.40)	<.0005
Fibrinogen γ’/fibrinogen	0.31 (0.27-0.35)	0.32 (0.28-0.36)	.57	0.25 (0.22-0.27)	.005

BMI, body mass index.

a Mean values (95% CIs) before and 6 months after bariatric surgery were compared with a paired *t*-test.

b Mean values (95% CIs) in individuals before bariatric surgery and in controls were compared with a paired *t*-test.

When comparing 19 medication-free individuals before surgery with 19 age- and sex-matched normal-weight individuals, significant differences between the groups were observed, with lower values in the control group for all 4 variables, as presented in Table 2.

## 4 Discussion

In the present study, we generated mAbs against the C-terminal fibrinogen γ’ chain and performed a series of experiments to develop an ELISA for plasma concentrations of fibrinogen γ’. Specificity of the fibrinogen γ’ mAb was demonstrated by western blotting, showing reactivity against the γ’ chain only. This was followed by a systematic validation of the assay, where serial dilutions of fibrinogen γ’ (P2 Fib) and citrated plasma verified equally well recognition of standard and analyte. Accuracy, measured by spike and recovery, revealed a mean recovery of 88%. Sensitivity demonstrated low values of LOD and LOQ at 0.003 mg/mL and 0.014 mg/mL, respectively, and precision estimates were 3% for repeatability SD and 7% for within-laboratory SD. The fibrinogen γ’ reference interval was 0.25 to 0.80 mg/mL.

The validated assay was applied to plasma samples from 60 individuals before and 6 months after bariatric surgery, showing a decrease in fibrinogen γ’ but an unchanged ratio of fibrinogen γ’ to fibrinogen. Levels of fibrinogen γ’ and the fibrinogen γ’/fibrinogen ratio were lower in 19 normal-weight individuals compared with those in 19 individuals with obesity.

Almost 20 years ago, Uitte de Willige et al. [35] published an ELISA measuring fibrinogen γ’ based on another monoclonal anti-fibrinogen γ’ antibody, 2.G2.H9. This assay was partly validated with no measures of sensitivity and accuracy and no explanation of how they measured the analytical variation of the assay. In 2010, a well-characterized 2.G2.H9-based fibrinogen γ’ ELISA was published by Lovely et al. [22], although not including measures of accuracy and sensitivity. When comparing our ELISA to these previously published assays, our assay was validated with respect to spike and recovery and LOD. Importantly, our ELISA showed the lowest precision estimates on both repeatability and within-laboratory variation and is a valuable supplement to existing assays based on the 2.G2.H9 anti-fibrinogen γ’ antibody.

Lovely et al. [22] reported a reference interval of 0.09 to 0.55 mg/mL fibrinogen γ’ measured in a Framingham population (*n* = 2879) with no history of CVDs. Our ELISA yielded a reference interval of 0.25 to 0.80 mg/mL in Danish blood donors (*n* = 120). The mean ratio of fibrinogen γ’ to fibrinogen was 19%, which is slightly higher than the ratio of 10% to 15% reported in the general literature on fibrinogen γ’ [36]. The reason for these higher levels is unknown, and mean BMI (25.6 kg/m^2^) and age (29.8 years) in our blood donor cohort were lower than BMI (27.5-28.9 kg/m^2^) and age (58.8-63.5 years) in the Framingham cohort [22]. Thus, the higher fibrinogen γ’ levels measured in our reference interval cannot be explained by a skewed cohort in the direction of higher BMI and age. A shortened version of fibrinogen γ’ named fibrinogen γ’^1-423P^, lacking the last 4 amino acids of the fibrinogen γ’ chain, was previously described [[37], [38], [39]], but the 2.G2.H9 mAb and our 19-5-1 mAb are expected to react equally with fibrinogen γ’^1-423P^. The specificity of our mAb was very high, as illustrated by the complete lack of reactivity toward fibrinogen γA and components in serum.

To our knowledge, this study is the first to show reduced levels of fibrinogen γ’ after bariatric surgery, and studies on causality of this effect might help understand a possible role of fibrinogen γ’ in reducing risk of arterial thrombosis after surgery. Our results demonstrated no surgery-induced effects on the fibrinogen γ’/fibrinogen ratio, indicating that after surgery, there is a similar reduction in fibrinogen γ’ and total fibrinogen.

We observed significantly higher concentrations of fibrinogen γ’ and a higher fibrinogen γ’/fibrinogen ratio in individuals with obesity compared with normal-weight individuals. The higher concentrations of fibrinogen γ’ could be explained by fibrinogen γ’ and total fibrinogen being known as acute phase reactants in the inflammatory process and be the result of the general inflammation associated with obesity [40]. The higher fibrinogen γ’/fibrinogen ratio might then suggest that obesity or inflammation affects the splicing of the fibrinogen γ-chain, thus producing relatively higher levels of fibrinogen γ’. In support of this, the fibrinogen γ’/fibrinogen ratio is elevated in the acute phase of CVD [20]. However, an interesting study in mice has indicated that variants of fibrinogen affect the development of obesity differently [26]. Thus, if fibrinogen γ’ is involved in the development of obesity, we tentatively speculate that the individuals with obesity participating in our study are prone to obesity due to the high relative amount of fibrinogen γ’. The effects of obesity on fibrinogen γ’ must, of course, be confirmed in larger studies.

A small study in children with obesity [27] reported an almost 2-fold higher concentration of fibrinogen γ’ in 15 children with obesity compared with 6 normal-weight children and found a decrease in fibrinogen γ’ levels when the children were losing weight after physical activity. These results are consistent with the results of our study, except that we observed a higher fibrinogen γ’/fibrinogen ratio in individuals with obesity compared with normal-weight individuals. Nevertheless, results on fibrinogen γ’ in children and adults should be compared with caution due to different hormonal and metabolic milieu in the 2 populations.

Strengths of our clinical study are that the effects of bariatric surgery on fibrinogen γ’ were measured in a paired design and included a normal-weight control group. Limitations are that bariatric surgery not only induces changes in body weight, but we observed no significant correlations between pre- and postsurgery changes in BMI and fibrinogen γ’ or the fibrinogen γ’/fibrinogen ratio. Individuals with obesity suffer from numerous comorbidities and receive various medications, some of which are discontinued after surgery. However, results were confirmed in medication-free patients, although the reduction in fibrinogen γ’ was only borderline significant (*P* = .06). This could be explained by a type II error due to the small sample size of medication-free patients (*n* = 19).

In conclusion, we produced a highly specific fibrinogen γ’ mAb, with no cross-reactivity toward fibrinogen γ, and used it in the setup of an ELISA for measurement of fibrinogen γ’ concentrations in plasma. The assay was thoroughly validated, demonstrating parallelism between calibrator and analyte, accuracy, sensitivity, as well as low estimates of repeatability and within-laboratory variation. The assay was applied on samples from 60 individuals with obesity before and 6 months after bariatric surgery, showing a reduced level of fibrinogen γ’ after surgery but unchanged fibrinogen γ’/fibrinogen ratio, indicating that after surgery, there is a similar reduction in fibrinogen γ’ and total fibrinogen. Levels of fibrinogen γ’ and the fibrinogen γ’/fibrinogen ratio were significantly lower in normal-weight individuals compared with individuals with obesity.
